# miR-154-5p Affects the TGF*β*1/Smad3 Pathway on the Fibrosis of Diabetic Kidney Disease via Binding E3 Ubiquitin Ligase Smurf1

**DOI:** 10.1155/2022/7502632

**Published:** 2022-01-27

**Authors:** Che Bian, Zhilin Luan, Haibo Zhang, Ruijing Zhang, Jing Gao, Yuxia Wang, Jia Li, Huiwen Ren

**Affiliations:** ^1^Department of Endocrinology and Metabolism, The Fourth Affiliated Hospital of China Medical University, Shenyang, China; ^2^Advanced Institute for Medical Sciences, Dalian Medical University, Dalian, Liaoning, China; ^3^Department of Gerontology, Xin Hua Hospital, Shanghai Jiaotong University School of Medicine, Shanghai, China

## Abstract

**Aim:**

The study is aimed at verifying miR-154-5p and Smurf1 combination in glomerular mesangial cells regulating TGF*β*1/Smad3 pathway-related protein ubiquitination in the model of diabetic rats renal tissues, primary mesangial cells, and cell lines.

**Methods:**

The diabetic SD rat model and high-glucose-cultured primary mesangial cells and cell lines were established. miR-154-5p mimic and inhibitor, Smurf1 siRNA, and TGF *β* 1/Smad3 inhibitor (SB431542) were pretreated to make the TGF*β*1/Smad3 pathway and ubiquitin changes. Fluorescence in situ hybridization was used for the miR-154-5p renal localization; molecular biological detection was adopted for cell proliferation, renal function, urine protein, and pathway proteins. After bioinformatics predicted binding sites, luciferase and Co-IP were used to detect miRNA and protein binding.

**Results:**

miR-154-5p was significantly increased and mainly concentrated in the glomerular of renal cortex in well-established diabetic rat renal tissues. Rno-miR-154-5p combined Rno-Smurf1 3′ UTR, while Smurf1 combined Smad3 directly. Meanwhile, miR-154-5p regulates TGF*β*1/Smad3-mediated cell proliferation via Smurf1 ubiquitination.

**Conclusion:**

miR-154-5p regulates the TGF*β*1/Smads pathway through Smurf1 ubiquitination and promotes the fibrosis process of diabetic kidney disease.

## 1. Introduction

Diabetic kidney disease (DKD), formerly known as diabetic nephropathy (DN), is one of the most common chronic microvascular complications of diabetes mellitus, leading to end-stage renal disease (ESRD) [1], involving various renal sections mainly of glomerulus [2], and regarding urinary albumin to creatinine ratio (UACR) as one of the effective noninvasive detection methods [3–5]. The TGF*β*1/Smads pathway is a classic DKD way to regulate the proliferation and fibrosis of mesangial cells [6, 7]. MicroRNA (miRNA) is a kind of noncoding RNA with the length of 18-25 highly conserved nucleic acids of which miR-154 is located in the miRNA-rich region of the 14q32 single-stranded chromosome in mammals [8], with one of the mature, miR-154-5p, indicating significant correlation with urine protein and fibrotic factors of diabetic patients in our previous studies [9, 10]. However, the specific mechanism of miR-154-5p regulating DKD has not been studied. Therefore, the purpose of this study is to detect the expression of miR-154-5p in various models *in vitro and in vivo* on the basis of the successful establishment of diabetic rat model in previous studies [11, 12], and to explore the specific molecular mechanism of miR-154-5p regulating DKD in glomerular mesangial cells through bioinformatics prediction and verification.

## 2. Materials and Methods

### 2.1. Reagents

All the reagents were listed in Table 1. The siRNAs targeting Smurf1, miR-154-5p mimics, inhibitors, and plasmid vectors using psiCHECK 2.0 Vector System for the construction of target gene 3′ UTR, as well as their corresponding negative controls, were designed and synthesized by GenePharma, Shanghai, China. These reagents were transfected into cells using Lipofectamine™ 2000 Transfection Reagent (Invitrogen, USA) according to the manufacturer's instructions. The TGF *β* 1/Smad3 pathway inhibitor, SB431542 (#14775, 10 nmol/l) was used to pretreat target cells as described in previous studies [13]. After the transfection and pretreatment, cells were collected and stored in liquid nitrogen for the follow-up experiments.

### 2.2. Animal Modeling

Sprague-Dawley (SD) rats (SPF grade, 7 weeks old, 180-220 g, purchased from Beijing Vital River Laboratory Animal Technology Co., Ltd.), were fed with free water and food at a constant temperature (23 ± 2°C) and humidity (50-60%), with a day/night cycle of 12/12 h. The experiments were conducted from 9:00 a.m. to 11:00 a.m. daily to prevent circadian rhythm from influencing the results. All experimental protocols for animals have been approved by the Institutional Animal Care and Use Committee (IACUC) of China Medical University (Approval No. 2021115).

Rats were randomly assigned after one week adaptive feeding as the diabetic nephropathy group (DN, *n* = 10 rats): fed with continuous high-fat diet (D12492, energy ratios of fat, carbohydrate, and protein as 60 : 20 : 20 kcal%, total energy of 5.24 kcal/gm, Research Diets, USA) and multiple injections with a low dose of streptozotocin (STZ, 35 mg/kg, cold 0.1 m sodium citrate buffer pH 4.5, S0130, Sigma-Aldrich, USA) after 8 weeks high-fat diet induction and the normal control group (NC, *n* = 10 rats): fed with control diet (D12450J, energy ratios of fat, carbohydrate, and protein as 10 : 20 : 20 kcal%, total energy of 3.85 kcal/gm, Research Diets, USA) and multiple injections with sodium citrate buffer as placebo. The specific modeling methods refer to our previous studies [11, 12].

### 2.3. Biochemical Detection

The intraperitoneal glucose tolerance test (IPGTT) and insulin release test (IRT) were performed for the detection of rat blood glucose and insulin levels. After 12-16 h starvation, rats were intraperitoneally injected with 2 g/kg glucose, and blood glucose testing strips (OneTouch® Ultra, LIFESCAN, USA) were used for the detection of rat blood samples at 0, 5, 10, 30, 60, and 120 min, respectively. The enzyme-linked immunosorbent assay (ELISA) was used to measure rat insulin levels with the rat insulin ELISA kit (INS, CSB-E05070r, CUSABIO, USA). Homeostatic model of insulin resistance index (HOMA-IR) and insulin sensitivity index (ISI) were used to evaluate the insulin resistance and sensitivity, and GraphPad software was used to calculate the area under curve of glucose and insulin. When the metabolic cages were conducted, urine samples were collected and levels of urinary albumin with the rat microalbuminuria ELISA kit (MAU/ALB, CSB-E12991r, CUSABIO, USA) were detected. Chemiluminescence was used to detect creatinine (Cr) and blood urea nitrogen (BUN). Urinary albumin/creatine ratio (UACR, MAU/Cr ratio) is used for the detection of urinary protein.

### 2.4. Glomerular Isolation and Primary Mesangial Cell Culturing

Rat glomerulars were isolated from 2-3 rat renal tissues by dissecting renal cortex and medulla, digested with collagenase IV, subjected to serial sieving by cell strainer (70 and 100  *μ* m, BD Falcon), and rinsed with Hanks' balanced salt solution (Thermo Scientific™). Then, the glomerular was cultured with 10% FBS DMEM (Gibco™) supplemented with 100 U/ml penicillin-0.1 mg/ml streptomycin (Gibco™) or stored for subsequent experiments. Primary mesangial cells (PMCs) climbed out of the glomerular after 2-3 days of culturing and were first passaged after 5-7 days and then were cultured with the normal culturing process. Cells in their 3^rd^ to 4^th^ generation at a ratio of 2 × 10^5^ viable cells/well in 6-well culture plates were used for subsequent experiments [14].

### 2.5. Primary Proximal Tubular Epithelial Cell Culturing

Mouse primary proximal tubular epithelial cells (PPTCs) were extracted from renal tissues of 4-6 mice by dissecting renal cortex as well as inner and outer medulla according to the previous literature [15]. Then, the cortex and outer medulla were digested with collagenase IV, subjected to serial sieving by cell strainer (40  *μ* m, BD Falcon), rinsed with Hanks' balanced salt solution (Thermo Scientific™), layered with Percoll® (Sigma-Aldrich), and cultured with 10% FBS DMEM/F12 (Gibco™) supplemented with insulin-transferrin-sodium selenite media supplement (Sigma-Aldrich), 1 mM hydrocortisone (MedChemExpress®), 50 mM vitamin C (MedChemExpress®), and 100 U/ml penicillin-0.1 mg/ml streptomycin (Gibco™). Cells at a ratio of 2 × 10^5^ viable cells/well in 6-well culture plates were used for subsequent experiments.

### 2.6. Cell Line Culturing and Treatment

Rat mesangial cells (RMCs, CRL-2573™) and 293T cells (293T/17 or HEK 293T/17, CRL-1126™) were purchased from American Type Culture Collection (ATCC®) and cultured with Dulbecco's modified Eagle's medium (DMEM) containing 10% fetal bovine serum (FBS, North American Source, Gibco) at 37°C with saturated humidity in 5% CO_2_ atmosphere. Cells in their 5^th^ to 9^th^ generation of logarithmic growth phase were inoculated into 25 cm^2^ culture flasks at a ratio of 1 × 10^6^/flask or 6-well tissue culture plates of 5 × 10^5^/well. After synchronization via starvation in Opti-MEM (Gibco, USA) for 24 h and confluency reached 70-80%, cells were cultured with normal glucose (NG, 5.5 mmol/l D-glucose), high mannitol (HM, 5.5 mmol/l D-glucose and 24.5 mmol/l mannitol), and high glucose (HG, 30 mmol/l D-glucose), respectively, for 24 h and were collected and stored at -196°C for the follow-up experiments.

### 2.7. Luciferase Reporter Assay

After using 293T validation, RMCs were inoculated into 24-well plates of 5 × 10^5^ cells/well beforehand and cotransfected with miR-154-5p mimic, inhibitor, pmirGLO-Smurf1-3′ UTR-WT or pmirGLO-Smurf1-3′ UTR-MUT reporter plasmids accordingly. After 24 h posttransfection, cells were lysed using passive lysis buffer (Promega) and the luciferase activity was measured with the Dual-Luciferase Reporter Assay System (Promega) and normalized to Renilla luciferase activity, respectively. Experiments were performed in triplicate.

### 2.8. Fluorescence In Situ Hybridization (FISH)

FISH assays were performed using fluorescent in situ hybridization kit (Servicebio, China) according to the protocol. FAM-double-labeled miR-154-5p probe were designed and synthesized by Servicebio (China). Tissues were first fixed in 4% formaldehyde for 15 min, then permeabilized in PBS containing 0.5% Triton X-100 at 4°C for 30 min, and prehybridized at 37°C for 30 min in prehybridization solution. After that, probes were added in the hybridization solution and incubated with the tissue sections at 37°C overnight in the dark. The next day, the tissue sections were counterstained with DAPI for nuclear and imaged and then measured by a digital microscope application, CaseViewer (3DHISTECH Ltd.), for supporting histopathological diagnostic workflow and the microscope examination process in bioscience.

### 2.9. miRNA Real-Time PCR Assay

The miRcute miRNA Isolation Kit (DP501, Tiangen Biotech) was used for the isolation of miRNA and miRcute miRNA First-Strand cDNA Synthesis Kit (KR201, Tiangen Biotech) for the reverse transcription from miRNA into cDNA. The reverse transcriptional reaction process was 26°C for 20 min, 42°C for 40 min, and 85°C for 10 min holding. The miRcute miRNA qPCR Detection Kit (SYBR Green, FP401, Tiangen Biotech) was used to amplify the PCR reaction via Thermal Cycler Dice Real Time System (TaKaRa). The primer sequences of rno-miR-154-5p (designed and synthesized by GenePharma) were shown in Table 1. The PCR reaction system was 20 *μ*l containing 2 *μ*l cDNA with the response procedures: after initial degeneration at 95°C for 3 min, degeneration at 95°C for 12 s, annealing at 62°C for 40 s, and extension at 72°C for 30 s with 40 circles. The CT value was read for dissolution curve analysis with U6 snRNA (Tiangen Biotech) for internal standardization, while 2^-*ΔΔ*CT^ method was used to calculate the relative expression.

### 2.10. Co-immunoprecipitation

The EZ Magna RNA immunoprecipitation Kit (Millipore, USA) was used following the guidelines. Briefly, RMCs were lysed in RIP lysis buffer. Magnetic beads were preincubated with antibodies for 30 min at room temperature and the cell lysates were immunoprecipitated with beads for 6 h at 4°C. Then, protein was purified and detected by western blotting. Antibody information of Smurf1 and Smad3 is listed in Table 1.

### 2.11. Western Blotting

Protein samples were extracted with the lysis buffer containing the protease inhibitor, while total protein content was determined by the Pierce™ BCA Protein Assay Kit (Thermo Scientific™, USA). The SDS-PAGE electrophoresis was conducted after protein denaturation, and then, bands were transferred onto PVDF membranes according to a certain time. The membrane was blocked by 5% bovine serum albumin (BSA, Sigma Aldrich, USA) for 2 h and incubated with the corresponding concentration of primary antibodies (shown in Table 1) at 4°C overnight. After incubation of secondary antibodies at room temperature for 2 h, the Pierce™ ECL Western Blotting Substrate was used for membrane imaging via the imaging system (MicroChemi 4.2, Israel) with the detection of grey values by ImageJ 1.52i Java 1.8.0_172 (64-bit, National Institutes of Health, USA).

### 2.12. Cell Proliferation Assay

Cell samples were digested by trypsin and made into cell suspension. Appropriate cell density (5 × 10^3^-2 × 10^4^ cells/well) was inoculated on a 96-well plate for 24-72 h. Cell Counting Kit-8 (CCK-8, DOJINDO, Japan) Cell Proliferation and Cytotoxicity Assay Kit (Roche, USA) were used after cell adherence. CCK-8 detection solutions were added to 96-well plate for 2-4 h, while the absorbance of CCK-8 was read at 450 nm and 630 nm as reference by a full wavelength microarray (BioTek Power Wave XS).

### 2.13. Bioinformatics Analysis

Potential target mRNAs of miR-154-5p were predicted by the computer algorithm RNA22 V2 (https://cm.jefferson.edu/rna22/Interactive/). The mature miRNA sequences used by RNA22 V2 were downloaded from miRBase (http://www.mirbase.org/). Protein interaction analysis was performed using inBio_Map (v2016_09_12), IntAct Molecular Interaction Database, and STRING (version 11.0). All protein information was extracted from UniProtKB/Swiss-Prot database, and UbPred software was used to randomly predict the potential ubiquitination sites of proteins in the forest model. UbiBrowser and NetPath/NetSlim databases were used to verify ubiquitin ligase recognition characteristics and to locate known ubiquitin binding sites.

### 2.14. Statistical Analysis

The experiment was repeated more than three times under the same experimental conditions, and obtained data were statistically analyzed by SPSS 20.0 software. After testing each variable of normal distribution, normal distribution of measurement data was expressed with mean ± standard deviation ($\bar{x}\pm s$), while nonnormal distribution data with median (interquartile range). Students' *t*-test (between two groups) and one-way analysis of variance (ANOVA, among three or more groups) were used for the comparison followed by multiple comparison using the least square method *t*-test for homogeneity of variance and Tamhane's T2 test for heterogeneity of variance. *P* < 0.05 was considered of statistically significance with two tails.

## 3. Results

### 3.1. Changes of miR-154-5p and Pathway in Animal Models

**Figure 1:**
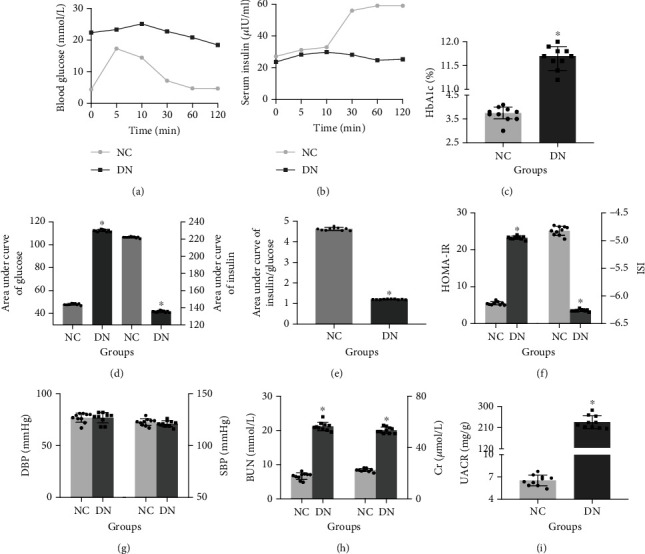
Physiological indexes in diabetic rats. Time-dependent curve in intraperitoneal glucose tolerance test (a) and insulin release test (b), HbA1c levels (c), area under curve of blood glucose (d left) and insulin (d right), area under curve ratio of insulin to blood glucose (e), HOMA-IR and ISI (f), DBP/SBP (g), renal function (h), and UACR (i). NC: normal control, DN: diabetic nephropathy. ^∗^vs. NC, *P* < 0.05. *n* = 10 rats/group.

We successfully established the diabetic rat model induced by high-fat diet and STZ based on previous studies. Physiological indexes indicated that the IPGTT overall level curve, HbA1c, area under curve of glucose, and HOMA-IR significantly increased, while the area under curve of insulin, area under curve of insulin/glucose ratio, and ISI significantly decreased, but the IRT curve does not appear to have an obvious peak in the DN group. In addition, DBP and SBP had no significant changes, excluding the influence of blood pressure. Renal function (BUN and Cr) and urinary protein (UACR) were significantly increased (Figure 1).

**Figure 2:**
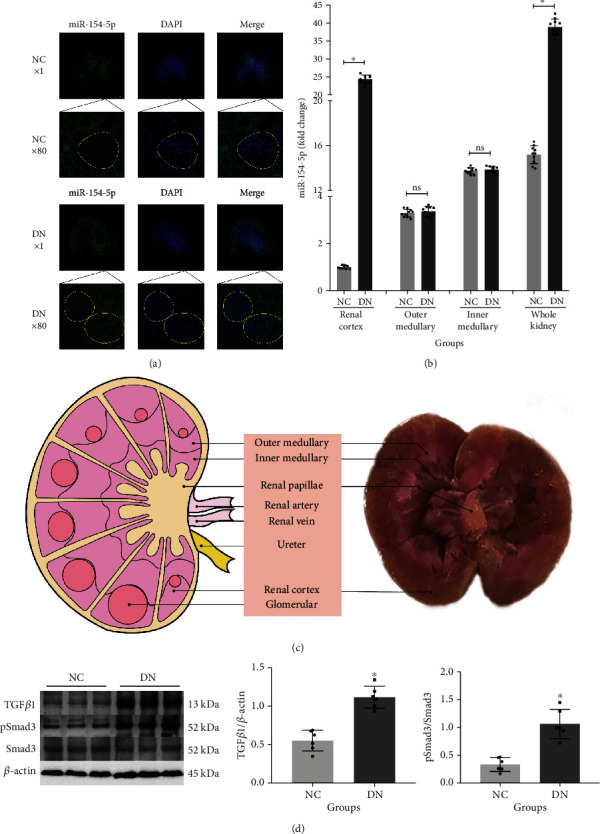
Expression of miR-154-5p in renal tissues. FISH detection for miR-154-5p localization in the kidney; significant enrichment of miR-154-5p in the cortical region of diabetic rats, in particular, glomerular (a). miR-154-5p in the cortex, outer medullary, inner medullary, and total kidney; homogenized semiquantified according to the NC group in the renal cortex (b). Microanatomy of the kidney (c). Protein levels in the whole kidney (d). NC: normal control; DN: diabetic nephropathy. Yellow circles indicate the glomerular. ^∗^vs. NC, *P* < 0.05; ns: vs. NC, *P* > 0.05. *n* = 6 samples/group.

FISH results showed that the relative expression of miR-154-5p was significantly increased, and the localization of miR-154-5p was mainly concentrated in the renal cortex of well-established diabetic rat renal tissues. The enlarged graph showed that the expression of miR-154-5p was significantly enriched in the glomerular region (Figure 2(a)). To verify the results of the localization experiment, we performed fine anatomy of renal tissue. Real-time PCR results showed that the levels of the total renal tissues and cortex were significantly increased, while there were no significant changes in the inner and outer medulla (Figure 2(b)). The microdissection of the cortex and outer and inner medullary was seen in Figure 2(c). The results of the both experiments were consistent, indicating highly expression of miR-154-5p in the glomerulus. Moreover, pathway proteins (TGF*β*1 and pSmad3/Smad3) were significantly increased in the diabetic renal tissues (Figure 2(d)).

### 3.2. Changes of miR-154-5p and Pathway in Primary Cell and Cell Lines

**Figure 3:**
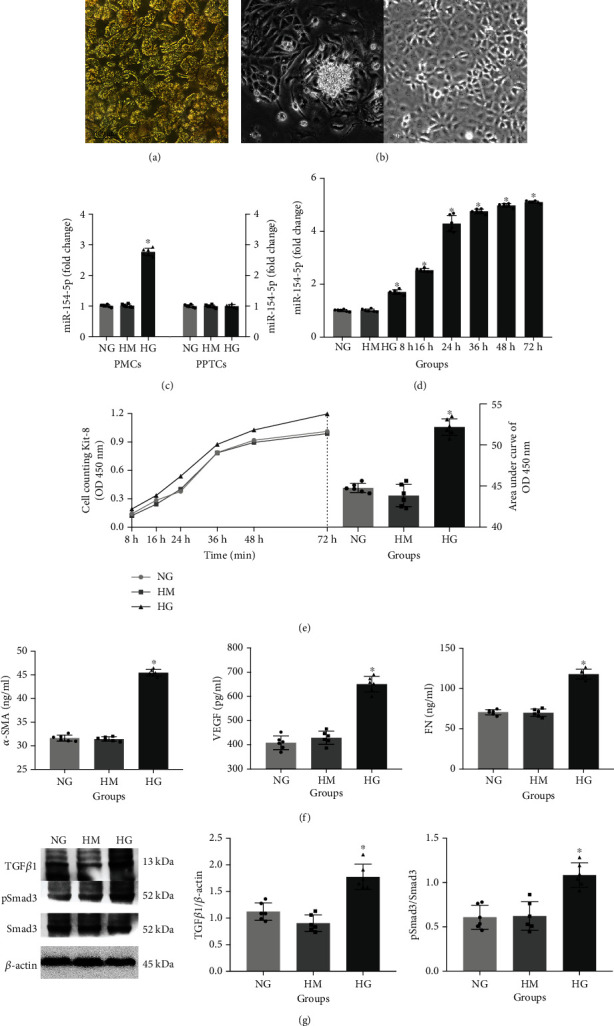
Expression of miR-154-5p in primary cells and cell lines. Rat glomerular isolation (a). Morphology extraction of primary mesangial cells (PMCs) and primary proximal tubular cells (PPTCs, b) with scale bar 50 *μ*m. miR-154-5p in high-glucose-cultured PMCs and PPTCs (c) as well as RMCs at different time points (d). CCK-8 cell proliferation in RMCs at different time points (e). Levels of VEGF, *α*-SMA, and FN in RMC supernatant (f). Protein expression in high-glucose-cultured RMCs (g). NG: normal glucose; HM: high mannitol; HG: high glucose. ^∗^vs. NC, *P* < 0.05. *n* = 6 samples/group.

We successfully isolated rat glomerulus (Figure 3(a)), and on the basis of successful culturing PMCs and PPTCs (Figure 3(b)), it was found that the expression of miR-154-5p in PMCs was significantly increased under high-glucose culturing, while with no changes in PPTCs (Figure 3(c)). Further study on the expression of miR-154-5p showed that the relative expression of miR-154-5p in mesangial cell lines, RMCs, under high-glucose culturing also showed a time-dependent increase and tended to be stable after high-glucose culturing for 24 h (Figure 3(d)). Compared with the NG group, the area under curve of CCK-8 OD at 450 nm and the levels of fibrotic factors in the NG group were significantly increased (Figures 3(e) and 3(f)). In addition, there were no significant changes in the above results of the HM group, excluding the effect of osmotic pressure. TGF*β*1 and pSmad3/Smad3 were both significantly increased in successfully established RMCs with a high-glucose culture (Figure 3(g)).

### 3.3. The Role of miR-154-5p Regulating the TGF*β*1/Smads Pathway

**Figure 4:**
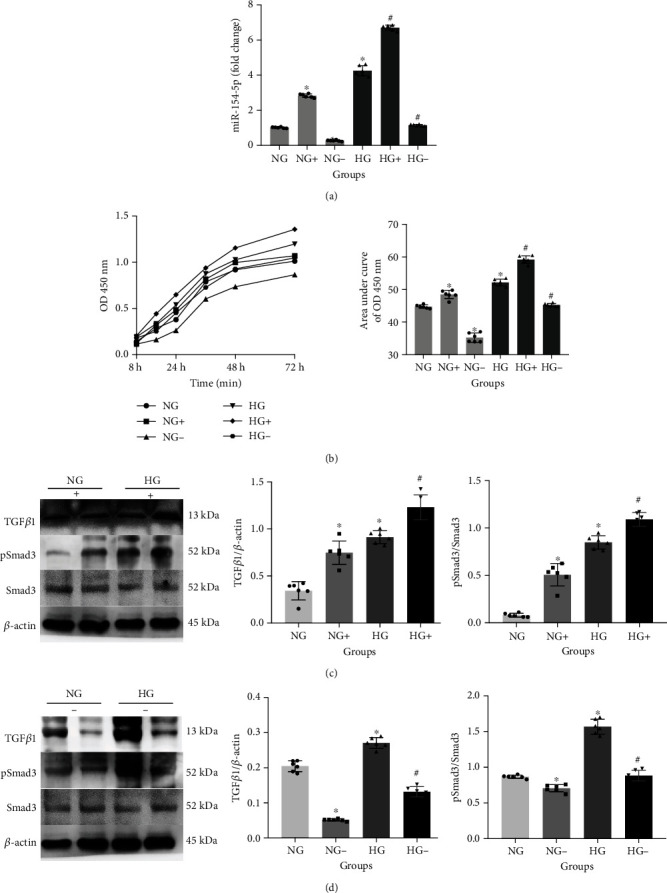
The role of miR-154-5p regulating the TGF*β*1/Smads pathway. Levels of miR-154-5p (a), CCK-8 cell proliferation (b), and protein expression levels (c, d) in RMCs with miR-154-5p mimic and inhibitor. NG: normal glucose; NG+ or - : normal glucose with mimic or inhibitor; HG: high glucose, HG+ or - : high glucose with mimic or inhibitor. ^∗^vs NG, *P* < 0.05; ^#^vs. HG, *P* < 0.05. *n* = 6 samples/group.

After the construction of the miR-154-5p mimic and inhibitor transfection, the expression of miR-154-5p proved that the transfection model was successfully constructed (Figure 4(a)). TGF*β*1 and pSmad3/Smad3 were significantly increased, and CCK-8 showed abnormal proliferation in normal- and high-glucose-cultured RMCs with the miR-154-5p mimic treatment. On the contrary, TGF*β*1 and pSmad3/Smad3 were significantly decreased in RMCs treated with the miR-154-5p inhibitor, and cell proliferation was decreased (Figures 4(b)–4(d)).

### 3.4. Verification of miR-154-5p Target Gene, Smurf1

**Figure 5:**
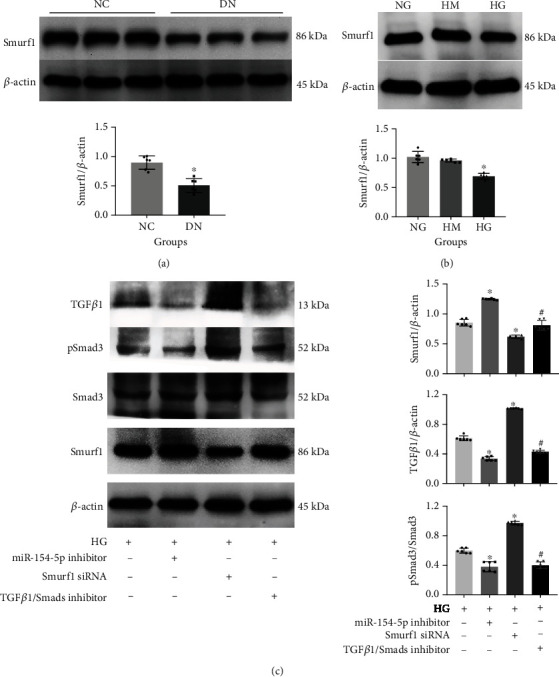
Indirect verification of the miR-154-5p target gene. Smurf1 in diabetic rats (a) and RMCs (b) cultured with high glucose. Smurf1 in RMCs with high glucose, miR-154-5p inhibitor, Smurf1 siRNA, and TGF*β*1/Smads pathway inhibitor (c). NC: normal control; DN: diabetic nephropathy; NG: normal glucose; HM: high mannitol; HG: high glucose. ^∗^vs. NC or NG, *P* < 0.05; ^#^vs HG, *P* < 0.05. *n* = 6 samples/group.

To further investigate the function of miR-154-5p, we used the RNA22 V2 computer algorithm to predict the target genes of human and rat miR-154-5p, and the mature miRNA sequences were downloaded from miRBase. The results of bioinformatics analysis showed that there was a target gene, Smurf1, binding with miR-154-5p in humans and rats with multiple binding sites (Figure S1), suggesting that humans and rats had highly similar binding patterns. Changes of Smurf1 were observed in renal tissues of diabetic rats and in vitro models cultured with high glucose (Figures 5(a) and 5(b)). After that, miR-154-5p inhibitor, Smurf1 siRNA and TGF*β*1/Smads pathway inhibitor were pretreated into RMCs cultured with high glucose, respectively. Levels of ubiquitin-related molecule, Smurf1, were significantly decreased in the Smurf1 siRNA group and significantly increased in the miR-154-5p inhibitor group. Smurf1 in the TGF*β*1/Smads pathway inhibitor group was reduced compared with the miR-154-5p inhibitor group. The expression of TGF*β*1 and pSmad3/Smad3 was significantly decreased in the miR-154-5p inhibitor and TGF*β*1/Smads pathway inhibitor groups and significantly increased in the Smurf1 siRNA group (Figure 5(c)).

### 3.5. Smurf1 Regulates Ubiquitination through Smad3

As a member of HECT family with E3 ubiquitin ligases, Smurf1 is a key enzyme that determines substrate specificity in the ubiquitin-modifying pathway. It can recognize ubiquitinated protein substrates and selectively regulate the degradation process of effector molecules Smads ubiquitination. The mechanism was seen in Figure S2A. Based on the analysis of known ubiquitination binding sites in the NetPath/NetSlim database, it was found that the known ubiquitination binding sites for Smurf1 were RhoA, Smad7, and TRI (Figure S2B). Smurf1 interacts with TGF*β*1 receptors, Smads, RhoA, Smurf2, and other Smurf1 proteins in inBio_Map (v2016_09_12) and IntAct Molecular Int analysis (Figure S3A). UbiBrowser database verified ubiquitin ligase recognition characteristics and found that Smurf1 had high ubiquitin binding ability to Smad2, Smad3, Smad4, and Smurf2, respectively (Figure S3B), and Smad3 had potential sites for binding to the C2 and HECT regions of Smurf1 (MH1: position 31-131, length 101; MH2: position 226-403, length 178), suggesting that Smurf1 and Smad3 may have a potential ubiquitination binding mode. Moreover, Smurf1 and Smad3 structures of humans and rats collected from the UniProtKB/Swiss-Prot database were compared. Sequence alignment results showed that the corresponding sequences in C2 and HECT regions of Smurf1 as well as the MH1 and MH2 regions of Smad3 were exactly the same in humans and rats (Figure S3C), indicating highly similar binding patterns in both humans and rats.

**Figure 6:**
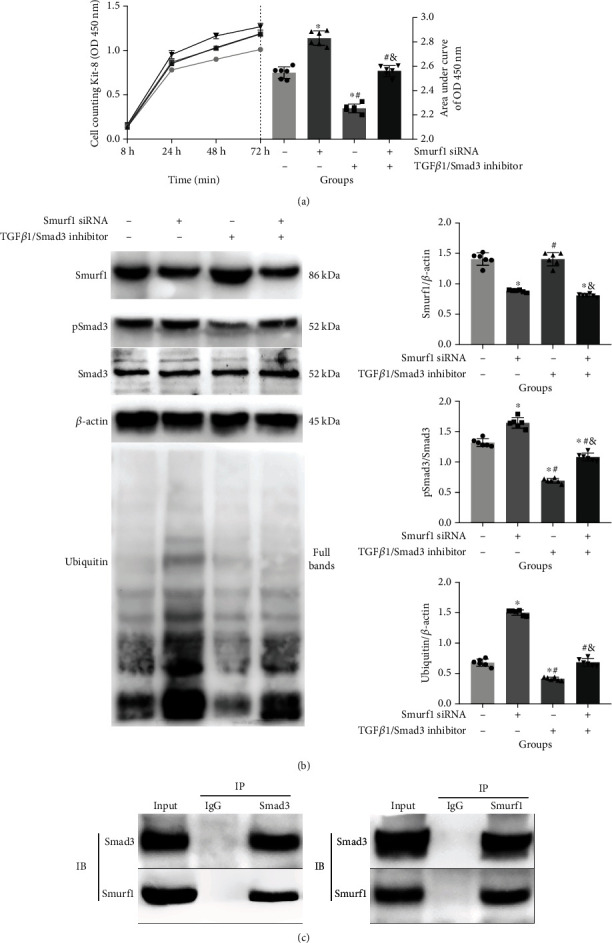
Smurf1 regulates ubiquitination through Smad3. CCK-8 cell proliferation (a) and related protein Smurf1, pSmad3/Smad3, and ubiquitin concentration (b). Co-IP verification for the combination of Smurf1 and Smad3 (c) after pretreatment with Smurf1 siRNA and TGF*β*1/Smad3 inhibitor, SB431542. ^∗^vs. Group 1, *P* < 0.05; ^#^vs. Group 2, *P* < 0.05; ^&^vs. Group 3, *P* < 0.05. *n* = 6 samples/group.

To verify the regulatory and binding effects of Smurf1 and Smad3, RMCs were treated with Smurf1 siRNA and TGF*β*1/Smad3 inhibitor, SB431542. Smad3 was found to restore Smurf1-induced pSmad3/Smad3 and ubiquitin expression (Figure 6(b)) as well as the abnormal cell proliferation detected by CCK-8 (Figure 6(a)). In addition, Co-IP validation found direct binding sites between Smurf1 and Smad3 (Figure 6(c)).

### 3.6. miR-154-5p Influences Smurf1-Mediated Ubiquitination of Smad3

**Figure 7:**
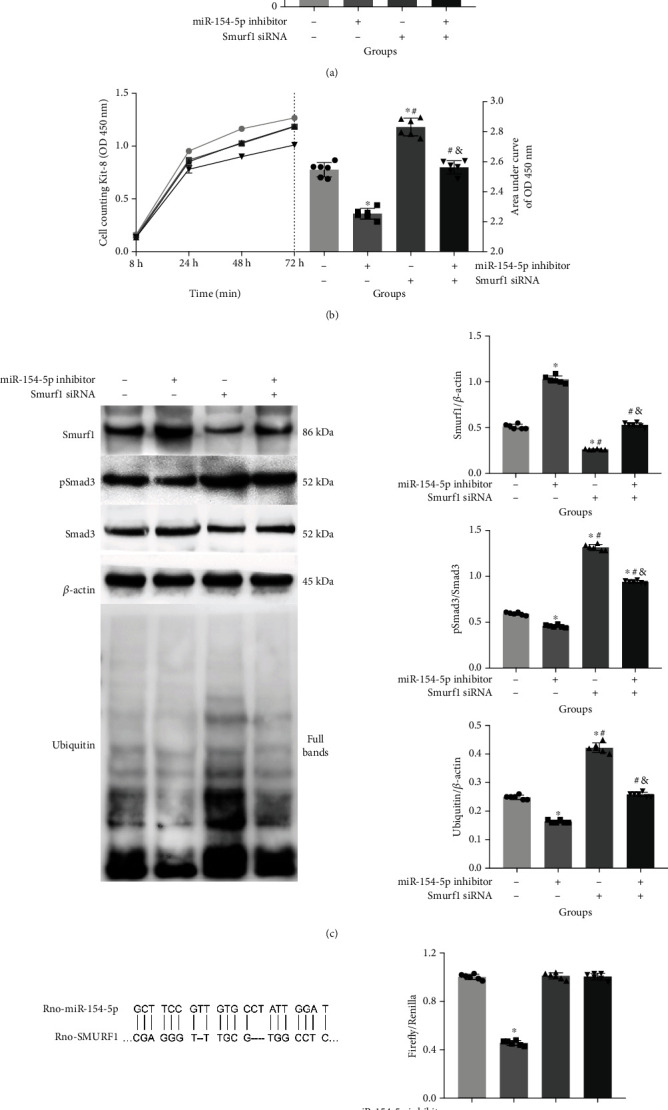
miR-154-5p influences Smurf1-mediated ubiquitination of Smad3. miRNA expression (a); CCK-8 cell proliferation (b); related protein Smurf1, pSmad3/Smad3, and ubiquitin concentration (c); luciferase verification for the binding of rno-miR-154-5p and rno-Smurf1 3′ UTR (d) after pretreatment with miR-154-5p inhibitor and Smurf1 siRNA. ^∗^vs. Group 1, *P* < 0.05; ^#^vs. Group 2, *P* < 0.05; ^&^vs. Group 3, *P* < 0.05. *n* = 6 samples/group.

To further verify the regulation and binding of miR-154-5p and Smurf1, RMCs were pretreated with the miR-154-5p inhibitor and Smurf1 siRNA, and the detection found that Smurf1 can reverse the regulation of miR-154-5p on pSmad3/Smad3 and ubiquitin expressions (Figures 7(a) and 7(c)) as well as the abnormal cell proliferation detected by CCK-8 (Figure 7(b)). In addition, Rno-miR-154-5p and Rno-Smurf1 3′ UTR were able to bind directly in the predictive analysis, and luciferase validation showed that Rno-miR-154-5p and Rno-Smurf1 3′ UTR produced direct binding (Figure 7(d)).

## 4. Discussion

Type 2 diabetes mellitus (T2DM) is a long-term metabolic disorder characterized by hyperglycemia, insulin resistance, and relative deficiency of insulin [16]. Diabetic kidney disease (DKD) is one of the most common chronic microvascular complications of T2DM, which can lead to end-stage renal disease (ESRD) and even renal failure [1, 17, 18] and increased death caused by cardiovascular events [19], which bring heavy economic burden to the society and family. The characteristic clinical manifestation of DKD is continuous and has slow development of proteinuria. In clinical practice, in addition to invasive renal biopsy as the gold standard for diagnosis, noninvasive urinary albumin to creatinine ratio (UACR) and glomerular filter rate detection are also used as the basis for diagnosis and classification [3–5]. DKD lesions can involve all parts of the kidney, including abnormal proliferation of glomerular mesangial cells, thickening of basement membrane, glomerular sclerosis, and podocyte loss in early stage, while renal tubular basement membrane thickening, tubular atrophy, renal interstitial inflammatory infiltration, and renal interstitial fibrosis were observed in the later stage [2]. Among them, abnormal proliferation of mesangial cells, renal interstitial fibrosis, and podocyte injury are important pathological processes of fibrosis, which run through the whole process of DKD disease, and have become an important biomarker to evaluate the progress of DKD.

Fibrosis is the core of high morbidity and mortality associated with DKD, and its production is mainly the result of multiple factors such as high glomerular filtration, increased advanced glycation end products, and reactive oxygen species, as well as the activation of renin-angiotensin-aldosterone system. Abnormal proliferation of rat mesangial cells (RMCs) is an important pathological change in the early stage of DKD fibrosis, and RMCs cultured in high glucose are a classic model for the study of DKD [20, 21]. The intracellular molecular pathways are believed related to mesangial cell proliferation and fibrosis including the activation of renin-angiotensin system, transforming growth factor *β*1 (TGF*β*1), monocyte chemotactic protein-1, connective tissue growth factor (CTGF), and fibronectin (FN), etc. [22–25], which can effectively assess the extent of renal injury and timely guide the clinical treatment of DKD [26–29].

MicroRNAs (miRNAs) are highly conserved noncoding RNAs with a length of 18-25 nucleic acids that regulate gene expression through incomplete complementary base sequences at the 3′ terminal untranslated region (UTR) of the target mRNA, thereby influencing multiple cellular processes ranging from growth and development to disease generation. Studies have shown that multiple families of miRNA clusters are involved in the pathogenesis of DKD, such as let-7 family, miR-21, and miR-377, which are involved in the proliferation and apoptosis of mesangial cells under the condition of high glucose, while miR-34a-5p, miR-184, and miR-1915-5p are associated with renal tubulointerstitial fibrosis [30, 31]. Our previous studies found that compared with the normal control group, serum miR-154-5p expression in type 2 diabetic patients was significantly increased and positively correlated with UACR, HbA1c, and fibrosis factors (CTGF, VEGF, FN and TGF*β*1) [9, 10], indicating that human circulating miR-154-5p was closely related to renal fibrosis. This is the first time that miR-154-5p has been found to be associated with DKD fibrosis so far, suggesting that miR-154-5p in circulating blood may be potentially associated with blood glucose and proteinuria regulating the process of DKD glomerular fibrosis. In the high-fat diet and STZ-induced diabetic rats, FISH and PCR were used to detect the expression of miR-154-5p, and the results consistently showed that miR-154-5p was highly expressed in the glomerular rather than proximal tubules of the cortex region. The glomerulus is also an important structure involved in the filtration of urinary protein, which is consistent with the results of our clinical trials.

The specific mechanism of DKD producing urine protein is thought to be related to early pathological changes of the abnormal proliferation in glomerular mesangial cells. Thus, the classic model of RMCs under high-glucose cultivation is adopted to explore DKD [20]. On the basis of our previous successful culturing RMCs [11, 12], we also successfully isolated the primary rat glomerular mesangial cells. Results by repeated detection of miR-154-5p showed that the expression of miR-154-5p was significantly decreased in both primary cells and cell lines pretreated by high-glucose culturing, and the cell proliferation activity was abnormally increased, which was consistent with the results *in vivo*, suggesting that miR-154-5p may be involved in the changes of glomerular mesangial cells in the early stage of DKD.

The important pathophysiological change of DKD is glomerular fibrosis, and the TGF*β*1 pathway is one of the main pathways regulating the proliferation and fibrosis of mesangial cells. TGF*β*1 binds to its membrane receptor, TGF*β* receptor 1 (TRI), and activates another receptor, TRII. TRII phosphorylates Smad2 and Smad3 in cells to form heteropolymer with Smad4. After nucleation, this complex binds to transcriptional coactivators or coinhibitors, thereby regulating the transcription of downstream target genes. In contrast, Smad7 binds to TRI and TRII and inhibits phosphorylation of Smad2 and Smad3, which in turn inhibits the TGF*β*1 pathway transmission. Smads are involved in the pathological process of the TGF*β*1 pathway in mesangial cell proliferation and fibrosis through both positive and negative regulatory effects [6, 7]. TGF*β*1 and pSmad3/Smad3 were significantly elevated in the diabetic animal model established in this study, which also suggested that the glomerular fibrosis of DKD was closely related to the TGF*β*1/Smads pathway.

miR-154 is located on the miRNA-rich region in the single-stranded chromosome of mammalian 14q32 [8], and the 5′ arm of the precursor miR-154 (sequence: 5′-UAG GUU AUC CGU GUU GCC UUC G-3, mature to form miR-154-5p) has been demonstrated to be controlled by a 200 kb differential methylation region (DMR) in the Dlk1-Gtl2 (rodents)/Dlk-Dio3 (human) structural domain upstream of miRNA clusters [32–34]. Previous studies have confirmed that miR-154-5p is associated with the fibrosis mechanism of several diseases [35–38]. The transcription factor binding analysis showed that miR-154 is rich in Smad3 binding elements (SBEs) that mediate the TGF*β*1 pathway during the growth and development stage [35, 39, 40], suggesting that miR-154 may be involved in the TGF*β*1/Smad3 signaling pathway.

To investigate the relationship between miR-154-5p and the TGF*β*1/Smads pathway, we treated RMCs with high glucose, miR-154-5p mimic, and inhibitor, respectively. The results showed that miR-154-5p could change the protein levels in the TGF *β* 1/Smads pathway and cell proliferation activity in RMCs. Moreover, in order to explore the regulatory mechanism of miR-154-5p involved renal fibrosis, we predicted the target genes of miR-154-5p indicating binding sites between human and rat miR-154-5p sequences and Smad ubiquitination regulatory factor 1 (Smurf1), suggesting that Smurf1 could bind with human and rat miR-154-5p. To verify the target genes of miR-154-5p, we detected the levels of ubiquitin-related molecule, Smurf1 in the successfully established models, and found that the expression was significantly decreased in diabetic rat renal tissues and high-glucose-cultured RMCs *in vivo and in vitro*.

As a member of HECT family, the E3 ubiquitin ligase, Smurf1, is a key enzyme that determines substrate specificity in the ubiquitin-modifying pathway. It can recognize ubiquitinated protein substrates and selectively regulate the ubiquitinated degradation process of effector molecules, Smads [41, 42]. The known ubiquitination binding sites were analyzed using the NetPath/NetSlim database, finding that RhoA, Smad7, and TRI were known ubiquitin binding sites of Smurf1. Protein interaction analysis showed that Smurf1 could interact with multiple Smads in the TGF *β* 1 pathway. By the comparison of Smurf1 and Smad3 structures in humans and rats, Smurf1 and Smad3 had high ubiquitination binding ability and similar binding mode with Smad3. The bioinformatics analysis provided the possibility for the binding research of both. It has been reported that Smurf1 participates in the fibrosis of DKD and obstructive nephropathy by inducing the apoptosis of glomerular mesangial cells [43] and regulating the epithelial-mesenchymal transition (EMT) induced by TGF*β* [44–46]. These results suggest that Smurf1 may regulate the TGF *β* 1/Smads pathway and participate in mesangial cell fibrosis, suggesting that Smurf1 may directly regulate Smad3 ubiquitination in a new way.

Rescue and luciferase experiments were used to prove the regulatory effect of miR-154-5p and Smurf1 directly and indirectly. Results showed that regulating miR-154-5p could save the abnormal proliferation and fibrosis of RMCs caused by Smurf1 regulating Smad3 ubiquitination, and miR-154-5p could bind to the Smurf1 3′ UTR. Meanwhile, rescue and Co-IP experiments were used to verify the regulation of Smurf1 and Smad3 ubiquitination directly and indirectly, indicating that regulation of Smurf1 could also rescue the abnormal proliferation and fibrosis of RMCs caused by Smad3 ubiquitination and that Smurf1 and Smad3 could also be directly combined. All the above experiments proved that miR-154-5p could regulate the levels of Smad3 by combining with Smurf1, thereby regulating the abnormal proliferation and fibrosis of glomerular mesangial cells and affecting the generation of proteinuria in the early DKD stage.

**Figure 8:**
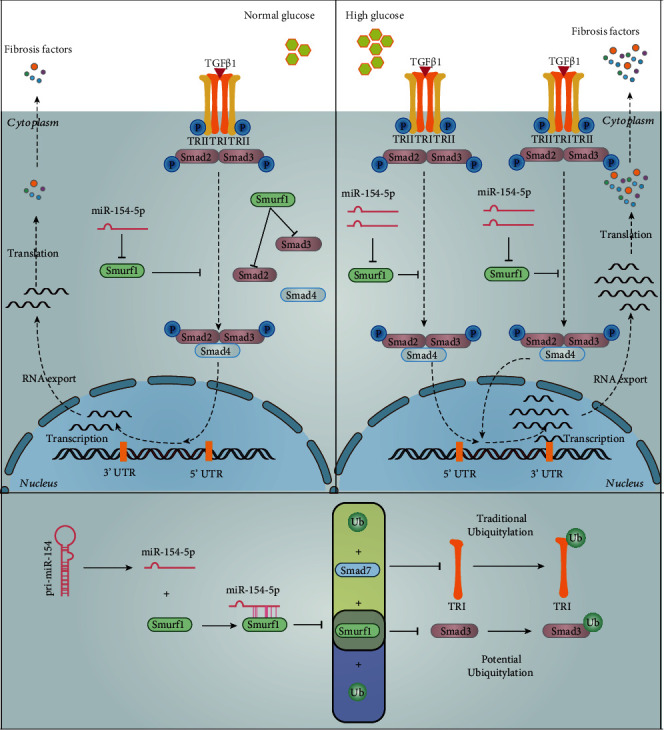
Possible mechanism. In the normal glucose state, TGF*β*1 activates TRI and TRII in small amounts, causing phosphorylated Smad2 and Smad3 to bind Smad4 as polymers, which enter the nucleus and participate in transcriptional regulation of fibrosis. Smurf1 inhibits TGF*β*1 signaling by regulating the classical pathway of Smad3 through TRI ubiquitination (Ub) via Smad7 or directly through the potential pathway of Smad3 ubiquitination. In the high glucose state, TGF*β*1/Smads pathway increases activation, causing more miR-154-5p to inhibit Smurf1, thus indirectly promoting TGF*β*1/Smads pathway activation and promoting the renal fibrosis process. The effect of lentivirus for the inhibitor of miR-154-5p can block this reaction thus indirectly alleviating diabetic kidney disease.

In conclusion, miR-154-5p can affect proliferation in glomerular mesangial cells via E3 ubiquitin ligase, Smurf1, regulating TGF*β*1/Smads pathway, thus affecting renal fibrosis of DKD. Hopefully, the inhibitor of miR-154-5p is expected to become a potential way for the DKD treatment. The possible mechanism is shown in Figure 8.

## Figures and Tables

**Table 1:** Information of reagents.

Names	Information
*Treatment*	
Rno-miR-154-5p mimic forward	5′ - UAG GUU AUC CGU GUU GCC UUC G -3′, GenePharma
Rno-miR-154-5p mimic reverse	5′ - AAG GCA ACA CGG AUA ACC UAU U -3′, GenePharma
Rno-miR-154-5p inhibitor	5′ - CGA AGG CAA CAC GGA UAA CCU A -3′, GenePharma
Rno-miR-negative control	5′ - CAG UAC UUU UGU GUA GUA CAA -3′, GenePharma
Smurf1 siRNA forward sequence	5′ - CAU AUC GCC AGA UCA UGA ATT -3′, GenePharma
Smurf1 siRNA reverse sequence	5′ - UUC AUG AUC UGG CGA UAU GTT -3′, GenePharma
Negative control siRNA forward	5′ - UUC UCC GAA CGU GUC ACG UTT -3′, GenePharma
Negative control siRNA reverse	5′ - ACG UGA CAC GUU CGG AGA ATT -3′, GenePharma
TGF*β*1/Smad3 inhibitor, SB431542	#14775, 10 nM for 24 h, dissolved in DMSO, Cell Signaling Technology
*Detection*	
Rno-miR-154-5p probe	5′-FAM-CGA AGG CAA CAC GGA TAA CCT A-FAM-3′ for FISH, GenePharma
Rno-miR-154-5p primer forward	5′ - CTG CCG TAG GTT ATC CGT G -3′, GenePharma
Rno-miR-154-5p primer reverse	5′ - AGA GCA GGG TCC GAG GAT -3′, GenePharma
U6 primer forward	5′ - CTC GCT TCG GCA GCA CA -3′, GenePharma
U6 primer reverse	5′ - AAC GCT TCA CGA ATT TGC GT -3′, GenePharma
TGF*β*1 primary antibody	Rabbit monoclonal antibody, ab215715, 44 kDa, 1 : 1000 for WB. Abcam
Smad3 primary antibody	Rabbit antibody, #9523, 52 kDa, 1 : 1000 for WB, 1 : 100 for IP, Cell Signaling Technology
pSmad3 primary antibody	Rabbit antibody, #9520, 52 kDa, 1 : 1000 for WB, Cell Signaling Technology
Smurf1 primary antibody	Mouse monoclonal antibody, sc-100616, 86 kDa, 1 : 200 for WB, 2 *μ*g/100 *μ*g total protein for IP, Santa Cruz Biotechnology
Ubiquitin primary antibody	Rabbit antibody, #3933, full bands, 1 : 1000 for WB, Cell Signaling Technology
*β*-Actin primary antibody	Rabbit antibody, #4970, 45 kDa, 1 : 1000 for WB, Cell Signaling Technology
IgG primary antibody	Rabbit antibody isotype control, #3900, for IP, Cell Signaling Technology
IgG primary antibody	Mouse antibody isotype control, #5415, for IP, Cell Signaling Technology
DAPI	For nucleus, #4083, for IF, Cell Signaling Technology
Anti-rabbit IgG	Anti-rabbit, #3678, for WB, Cell Signaling Technology
Anti-mouse IgG-HRP secondary antibody	Anti-mouse, #7076, for WB, Cell Signaling Technology
Anti-rabbit IgG-HRP secondary antibody	Anti-rabbit, #7074, for WB, Cell Signaling Technology

## Data Availability

The data used to support the findings of this study are included within the article.

## Supplementary Materials

Supplementary 1 Figure S1: target gene prediction of miR-154-5p. Above, prediction results of RNA22 V2 on the target gene (hsa_SMURF1) of miR-154-5p (hsa_miR_154_5p) in human. Below, prediction results of RNA22 V2 on the target gene (rno_SMURF1) of miR-154-5p (rno_miR_154_5p) in rats.

Supplementary 2 Figure S2: schematic diagram of Smurf1 ubiquitination. Schematic diagram of Smurf1 as E3 ubiquitination ligase for substrate recognition (A). Ub: ubiquitin, E1-E3: ubiquitin ligase; analysis of known ubiquitination binding sites (B).

Supplementary 3 Figure S3: prediction of protein interactions and ubiquitinated binding sites. Protein interaction analysis (A), the UbiBrowser database for verification of the ubiquitin ligase recognition characteristics (B), Smurf1 with high ubiquitin binding ability with Smad2, Smad3, Smad4, and Smurf2 (marked in red); human and rat Smurf1 and Smad3 sequence alignment (C); light red for the anchored alignment area, and blue for the initiation and termination sites amino acid sequences.
