# Supplementary material for: miR-154-5p Affects the TGFβ1/Smad3 Pathway on the Fibrosis of Diabetic Kidney Disease via Binding E3 Ubiquitin Ligase Smurf1
Source: Oxid Med Cell Longev. 2022 Jan 27;2022:7502632. doi: 10.1155/2022/7502632 (PMC8814716; doi:10.1155/2022/7502632)
Supplement: Supplementary 1 — Figure S1: target gene prediction of miR-154-5p. Above, prediction results of RNA22 V2 on the target gene (hsa_SMURF1) of miR-154-5p (hsa_miR_154_5p) in human. Below, prediction results of RNA22 V2 on the target gene (rno_SMURF1) of miR-154-5p (rno_miR_154_5p) in rats. [file 7502632.f1.pdf]

RNA22 v2 results

Resulted have computed and are shown below. If there are no results shown, it means your chosen parameters yielded no results.
Note: The p-value represents the likelihood that the target site loci is random. That is, a lower p-value represents a greater chance that the loci contains a valid MRE.

<table>
<tr><th>miR Name</th><th>transcript name</th><th>leftmost position of predicted target site</th><th>folding energy (in-Kcal/mol)</th><th>heteroduplex</th><th>p value</th></tr>
<tr><td>hsa_miR_154_5p
MIMAT0000452</td><td>hsa_SMURF1</td><td>985</td><td>-18.40</td><td>CAA TGG TGA --AA GGA TAA CCT C
|| | ||| ||| |||
GCT TCC GTT GTG CCT ATT GGA T</td><td>9.15E-2</td></tr>
</table>

<table>
<tr><th>miR Name</th><th>transcript name</th><th>leftmost position of predicted target site</th><th>folding energy (in-Kcal/mol)</th><th>heteroduplex</th><th>p value</th></tr>
<tr><td>rno_miR_154_5p
MIMAT0000856</td><td>rno_SMURF1</td><td>72</td><td>-18.30</td><td>CGC ATG GCG G--G CGG A--G GCC TG
|| | ||: : : ||| | : : ||| | :
GC-- TTC CGT TGT GCC TAT TGG AT</td><td>1.69E-1</td></tr>
<tr><td>rno_miR_154_5p
MIMAT0000856</td><td>rno_SMURF1</td><td>327</td><td>-16.70</td><td>CGA GGG T--T TGC G---- TGG CCT C
||| :|| : : :| | |: : |||
GCT TCC GTT GTG CCT ATT GGA T</td><td>3.45E-2</td></tr>
<tr><td>rno_miR_154_5p
MIMAT0000856</td><td>rno_SMURF1</td><td>1715</td><td>-17.10</td><td>AAA AGG CG-- CAA GGA --AG CCT G
||| |: || ||| |: ||| :
GCT TCC GTT GTG CCT ATT GGA T</td><td>3.06E-1</td></tr>
</table>
