# Supplementary material for: miR-154-5p Affects the TGFβ1/Smad3 Pathway on the Fibrosis of Diabetic Kidney Disease via Binding E3 Ubiquitin Ligase Smurf1
Source: Oxid Med Cell Longev. 2022 Jan 27;2022:7502632. doi: 10.1155/2022/7502632 (PMC8814716; doi:10.1155/2022/7502632)
Supplement: Supplementary 3 — Figure S3: prediction of protein interactions and ubiquitinated binding sites. Protein interaction analysis (A), the UbiBrowser database for verification of the ubiquitin ligase recognition characteristics (B), Smurf1 with high ubiquitin binding ability with Smad2, Smad3, Smad4, and Smurf2 (marked in red); human and rat Smurf1 and Smad3 sequence alignment (C); light red for the anchored alignment area, and blue for the initiation and termination sites amino acid sequences. [file 7502632.f3.pdf]

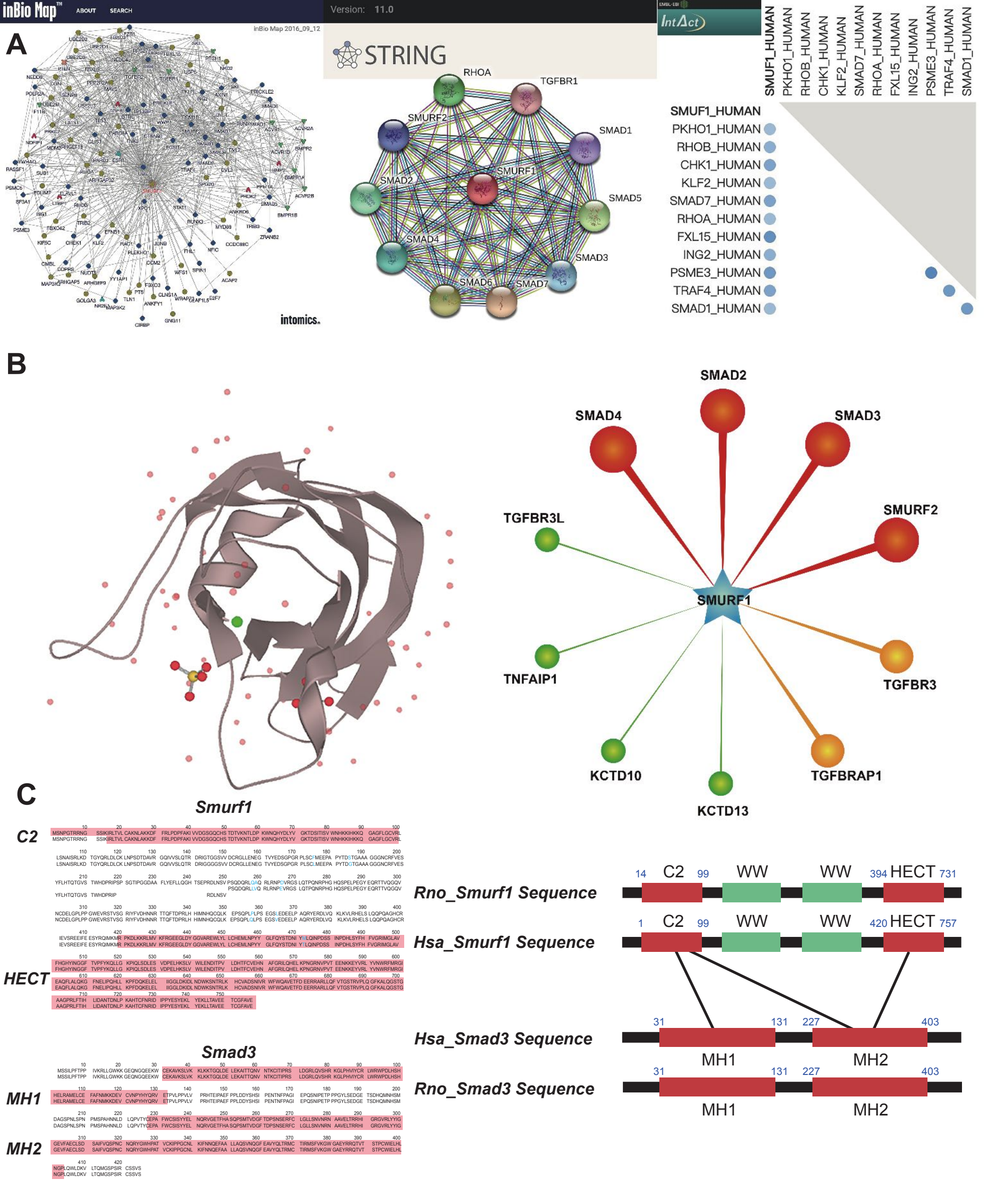
A
inBio Map™
ABOUT
SEARCH
inBio Map 2016_09_12
intomics.
Version: 11.0
STRING
RHOA
TGFBR1
SMURF2
SMAD1
SMURF1
SMAD2
SMAD5
SMAD4
SMAD3
SMAD6
SMAD7
IntAct
SMUF1_HUMAN
PKHO1_HUMAN
RHOB_HUMAN
CHK1_HUMAN
KLF2_HUMAN
SMAD7_HUMAN
RHOA_HUMAN
FXL15_HUMAN
ING2_HUMAN
PSME3_HUMAN
TRAF4_HUMAN
SMAD1_HUMAN
B
SMAD2
SMAD4
SMAD3
SMURF2
TGFBR3L
SMURF1
TNFAIP1
TGFBR3
KCTD10
KCTD13
TGFBRAP1
C
Smurf1
C2
HECT
Smad3
MH1
MH2
Rno_Smurf1 Sequence
Hsa_Smurf1 Sequence
Hsa_Smad3 Sequence
Rno_Smad3 Sequence
14 C2 99 WW WW 394 HECT 731
1 C2 99 WW WW 420 HECT 757
31 131 227 403
MH1 MH2
31 131 227 403
MH1 MH2
