# Supplementary material for: miR-154-5p Affects the TGFβ1/Smad3 Pathway on the Fibrosis of Diabetic Kidney Disease via Binding E3 Ubiquitin Ligase Smurf1
Source: Oxid Med Cell Longev. 2022 Jan 27;2022:7502632. doi: 10.1155/2022/7502632 (PMC8814716; doi:10.1155/2022/7502632)
Supplement: Supplementary 2 — Figure S2: schematic diagram of Smurf1 ubiquitination. Schematic diagram of Smurf1 as E3 ubiquitination ligase for substrate recognition (A). Ub: ubiquitin, E1-E3: ubiquitin ligase; analysis of known ubiquitination binding sites (B). [file 7502632.f2.pdf]

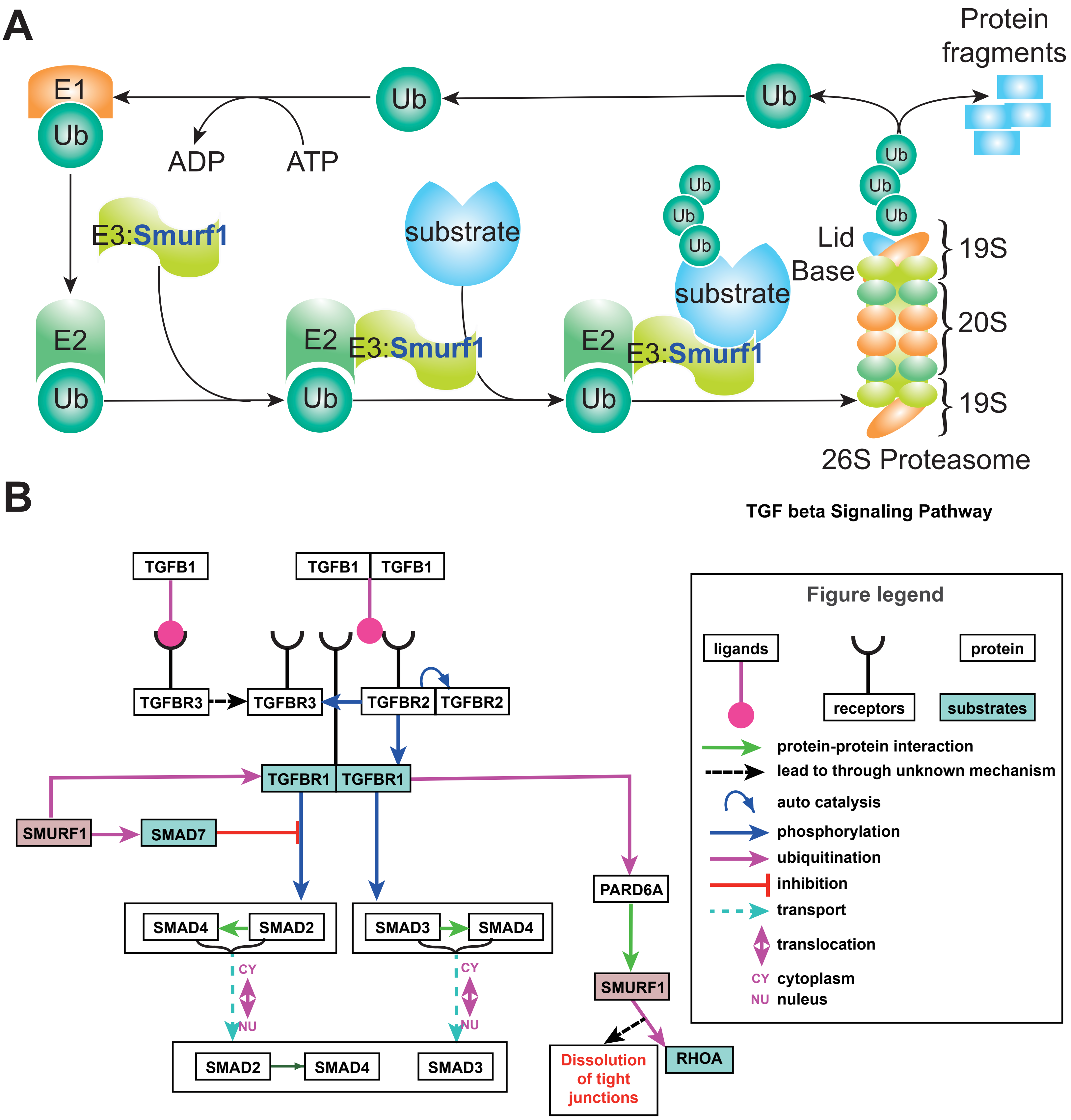
A
Protein fragments
E1
Ub
ADP
ATP
E3:Smurf1
substrate
E2
Lid
Base
19S
20S
26S Proteasome
B
TGF beta Signaling Pathway
TGFB1
TGFBR3
TGFBR2
TGFBR1
SMURF1
SMAD7
PARD6A
SMAD4
SMAD2
SMAD3
CY
NU
RHOA
Dissolution of tight junctions
Figure legend
ligands
receptors
protein
substrates
protein-protein interaction
lead to through unknown mechanism
auto catalysis
phosphorylation
ubiquitination
inhibition
transport
translocation
CY cytoplasm
NU nuleus
